# Supplementary material for: Integrated transcriptome landscape of ALS identifies genome instability linked to TDP-43 pathology
Source: Nat Commun. 2023 Apr 20;14:2176. doi: 10.1038/s41467-023-37630-6 (PMC10119258; doi:10.1038/s41467-023-37630-6)
Supplement: Supplementary file 1 — Supplementary Information [file 41467_2023_37630_MOESM1_ESM.pdf]

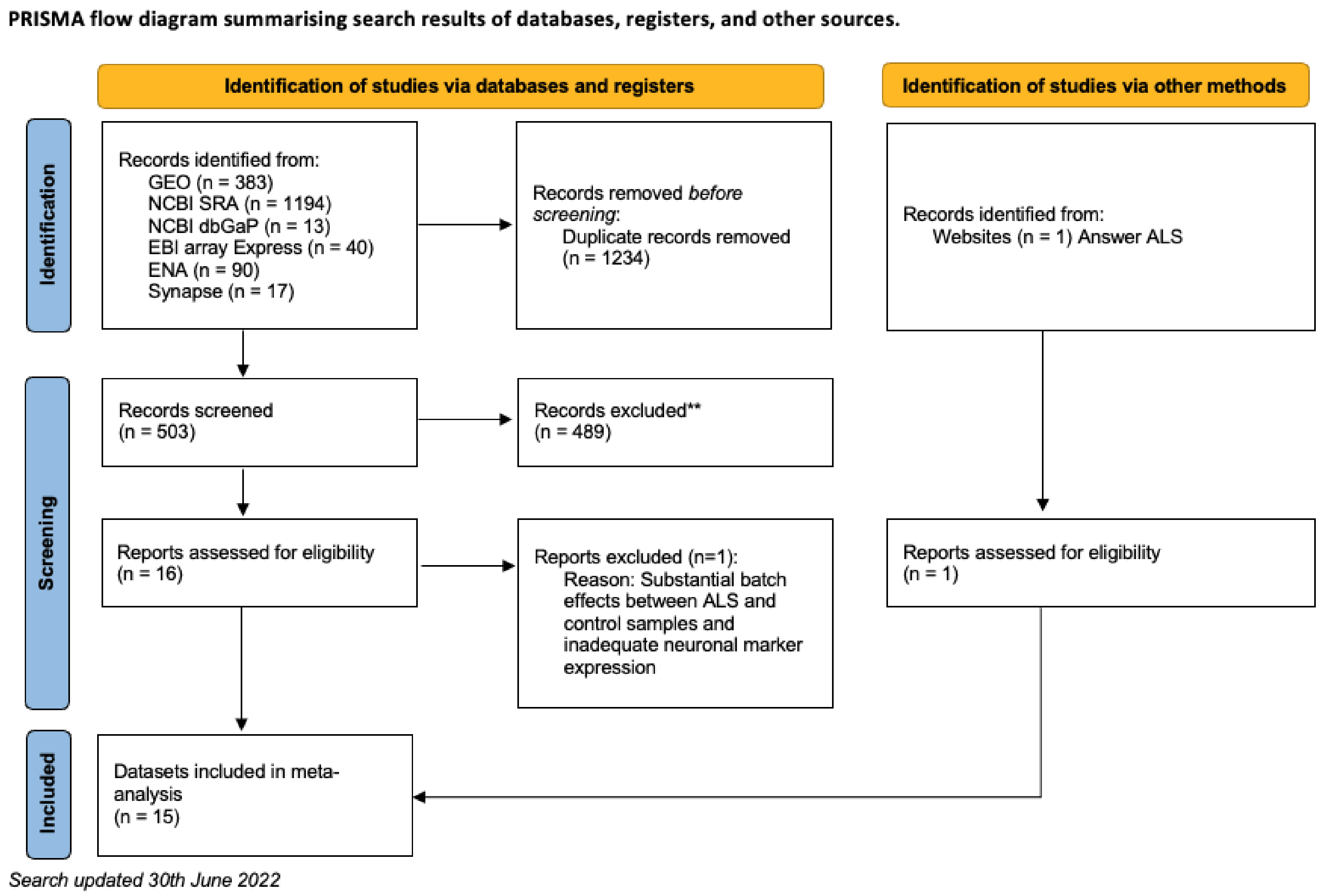

Supplementary Figure 1 PRISMA flow diagram of iPSMN database searches

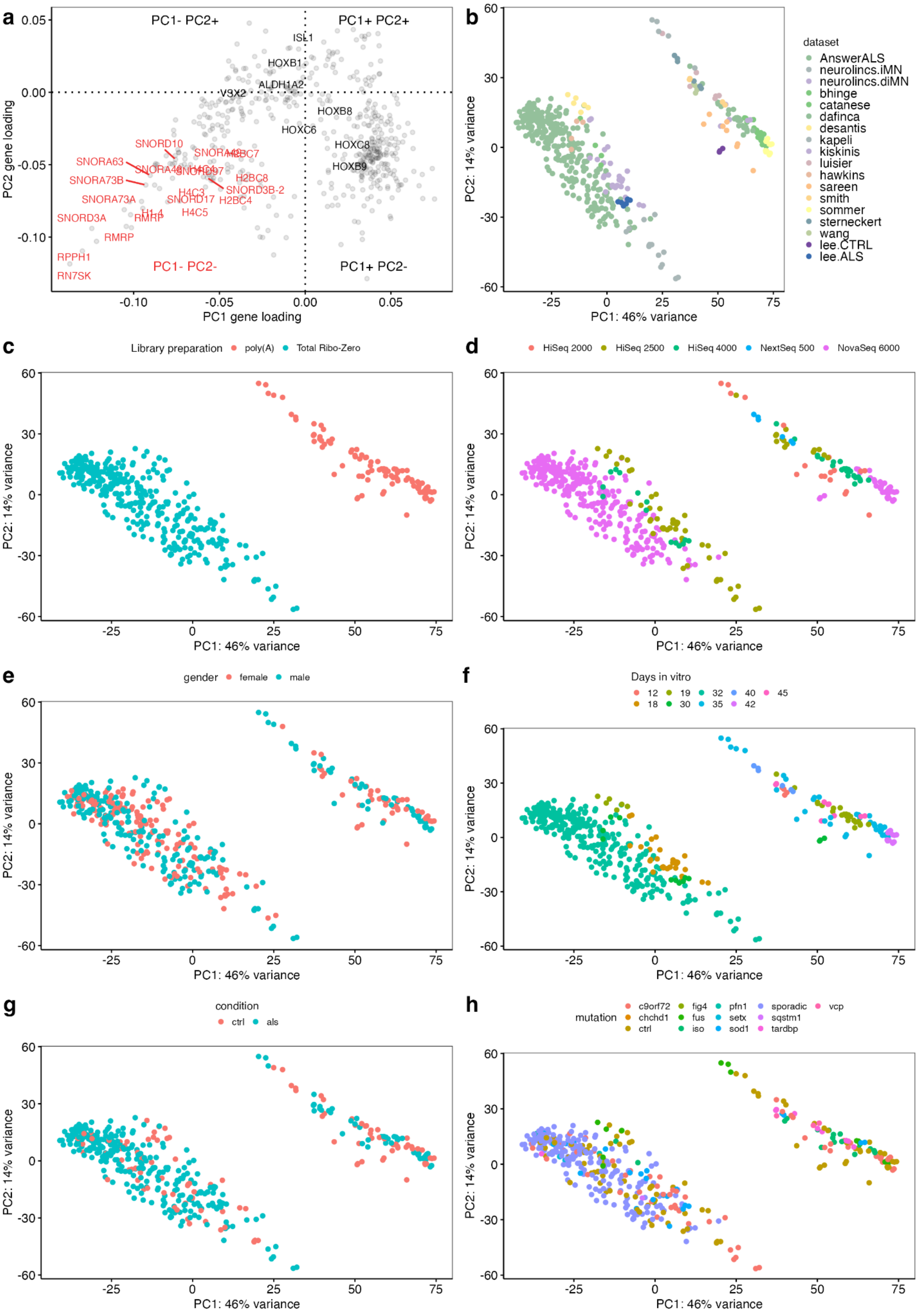

a
PC1- PC2+
PC1+ PC2+
PC1- PC2-
PC1+ PC2-
ISL1
HOXB1
ALDH1A2
V3X2
HOXB8
HOXC6
HOXC8
HOXB9
SNORD10
SNORA63
SNORA73B
SNORA73A
SNORD3A
RMRP
RPPH1
RN7SK
H2BC7
H2BC8
SNORD3B-2
H4C3
SNORD17
H2BC4
H4C5
H1-4
PC2 gene loading
PC1 gene loading
b
dataset
AnswerALS
neurolincs.iMN
neurolincs.diMN
bhinge
catanese
dafinca
desantis
kapeli
kiskinis
luisier
hawkins
sareen
smith
sommer
sterneckert
wang
lee.CTRL
lee.ALS
PC2: 14% variance
PC1: 46% variance
c
Library preparation
poly(A)
Total Ribo-Zero
d
HiSeq 2000
HiSeq 2500
HiSeq 4000
NextSeq 500
NovaSeq 6000
e
gender
female
male
f
Days in vitro
12
18
19
30
32
35
40
42
45
g
condition
ctrl
als
h
mutation
c9orf72
chchd1
ctrl
fig4
fus
iso
pfn1
setx
sod1
sporadic
sqstm1
tardbp
vcp

Supplementary Figure 2 Principal Component Analysis coloured by sample characteristics

a: Scatterplot of PC1 against PC2 individual gene loadings of the top 500 most variable genes. PC1- & PC2- loaded genes labelled red are histone and small non-coding RNAs, which represent non-polyadenylated transcripts. Genes labelled black are relevant to spinal motor neuron identity.

b-h: Principal component analysis of normalised transformed gene expression from iPSMN samples, coloured by (b) dataset, (c) RNA library preparation type, (d) sequencing instrument, (e) gender, (f) days in vitro, (g) disease status and (h) mutation. iso, isogenic correction.

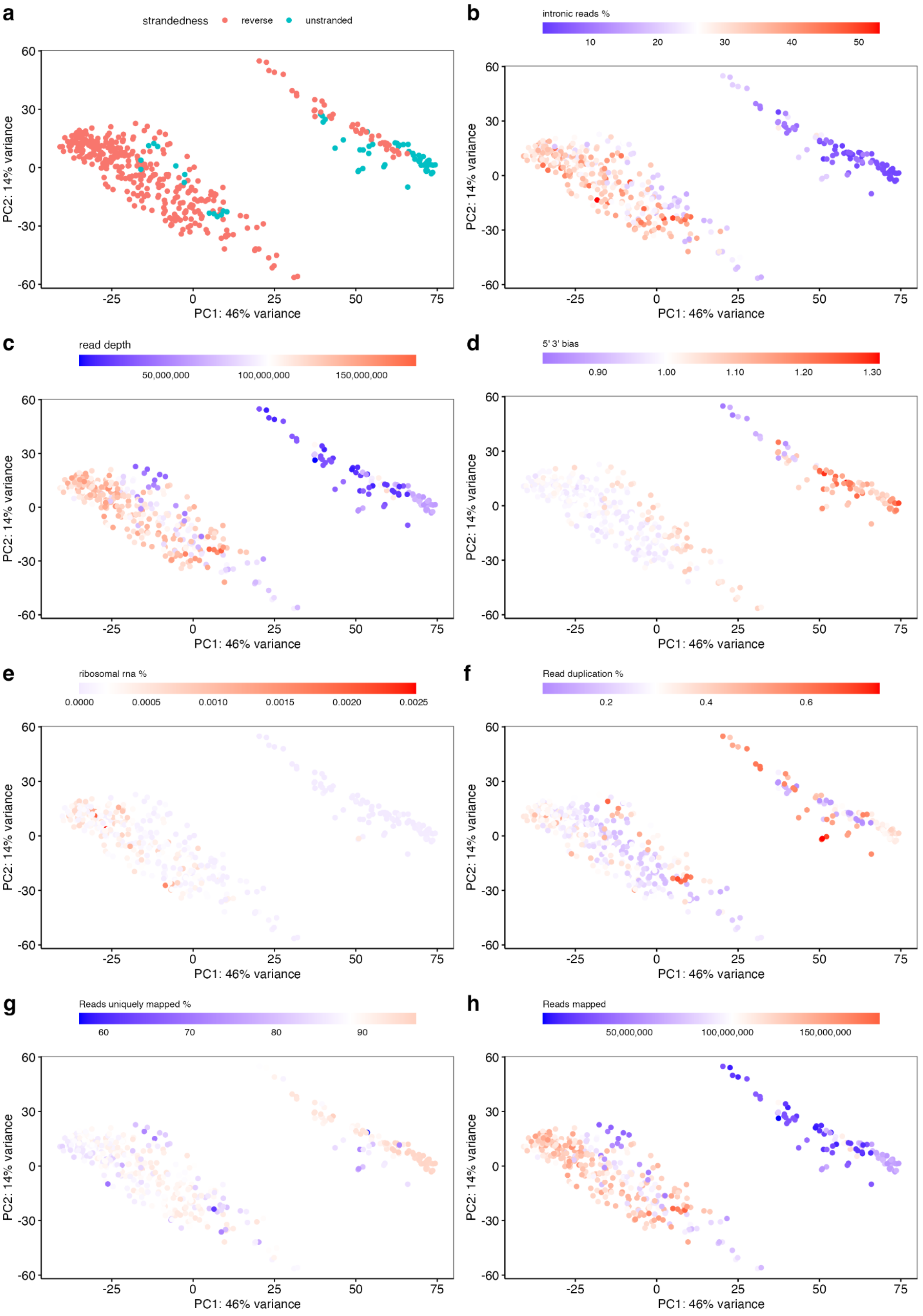
a
strandedness
reverse
unstranded
b
intronic reads %
10
20
30
40
50
c
read depth
50,000,000
100,000,000
150,000,000
d
5' 3' bias
0.90
1.00
1.10
1.20
1.30
e
ribosomal rna %
0.0000
0.0005
0.0010
0.0015
0.0020
0.0025
f
Read duplication %
0.2
0.4
0.6
g
Reads uniquely mapped %
60
70
80
90
h
Reads mapped
50,000,000
100,000,000
150,000,000
PC1: 46% variance
PC2: 14% variance
-25
0
25
50
75
60
30
0
-30
-60

Supplementary Figure 3 Principal Component Analysis coloured by QC metrics

a-h: Principal component analysis of normalised transformed gene expression from iPSMN samples, coloured by (a) strandedness, (b) intron read %, (c) read depth, (d) Picard read duplication %, (e) ribosomal RNA % biotype contamination, (f) Qualimap 5-3' bias, (g) STAR reads uniquely mapped % and (h) Samtools raw reads mapped. Scale bars are coloured from minimum (blue) to maximum (red) value with white representing the mean value.

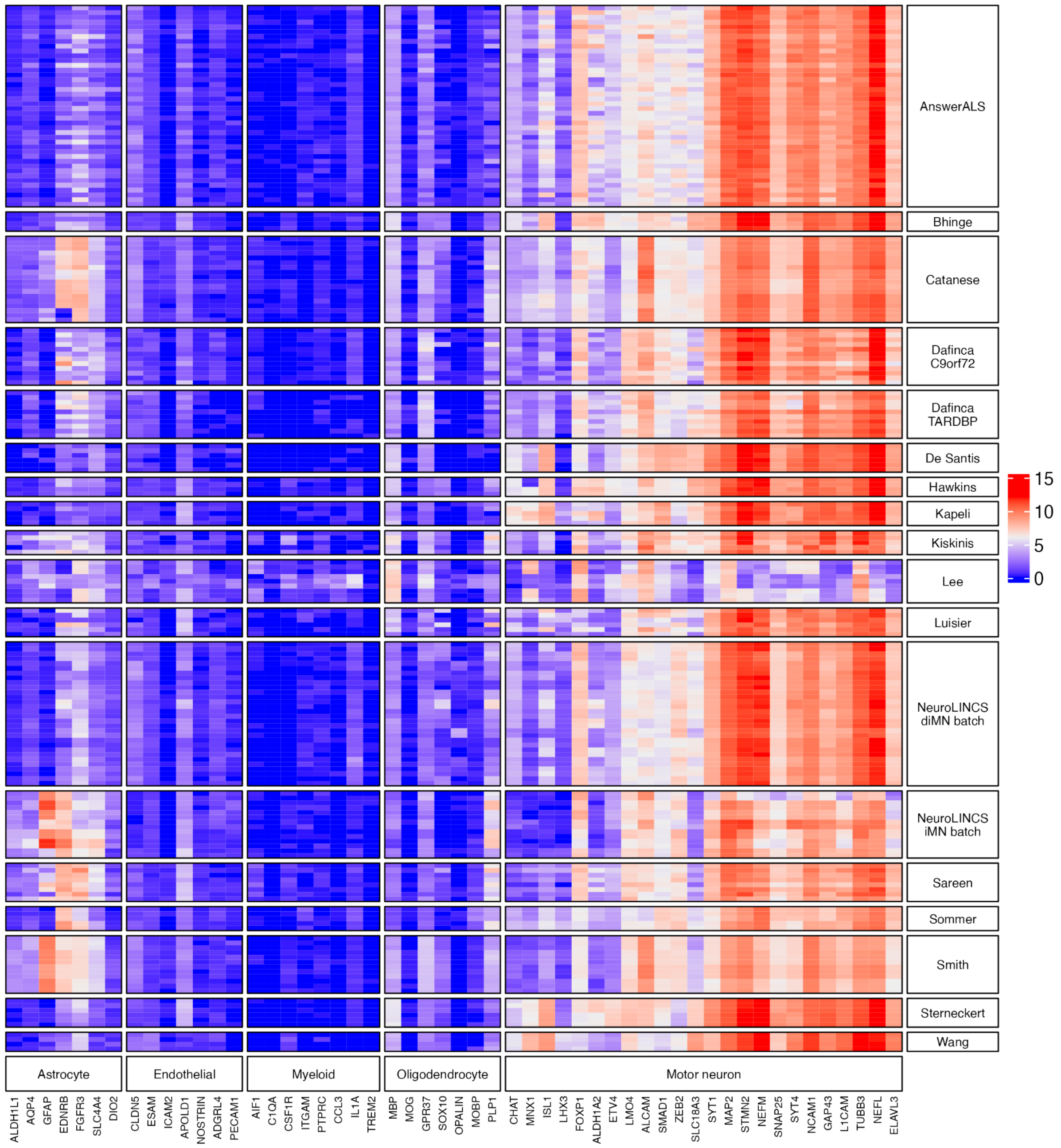

Supplementary Figure 4 Transcriptomic identities of iPSC-derived motor neurons

Heatmap of normalised gene counts for astrocytes, endothelial cells, myeloid cells, oligodendrocytes and motor neurons (columns) across iPSC-derived motor neuron datasets (rows). Blue represents low gene expression and red represents high expression, where 0 (dark blue) represents undetectable gene expression. The maximum (dark red) represents the greatest gene expression detected across all genes and samples plotted. To improve the visualisation of all datasets, for AnswerALS only 100 samples are plotted. Lee et al dataset is removed from the integrated analysis because of different library preparations between ALS and controls and poor neuronal marker expression.

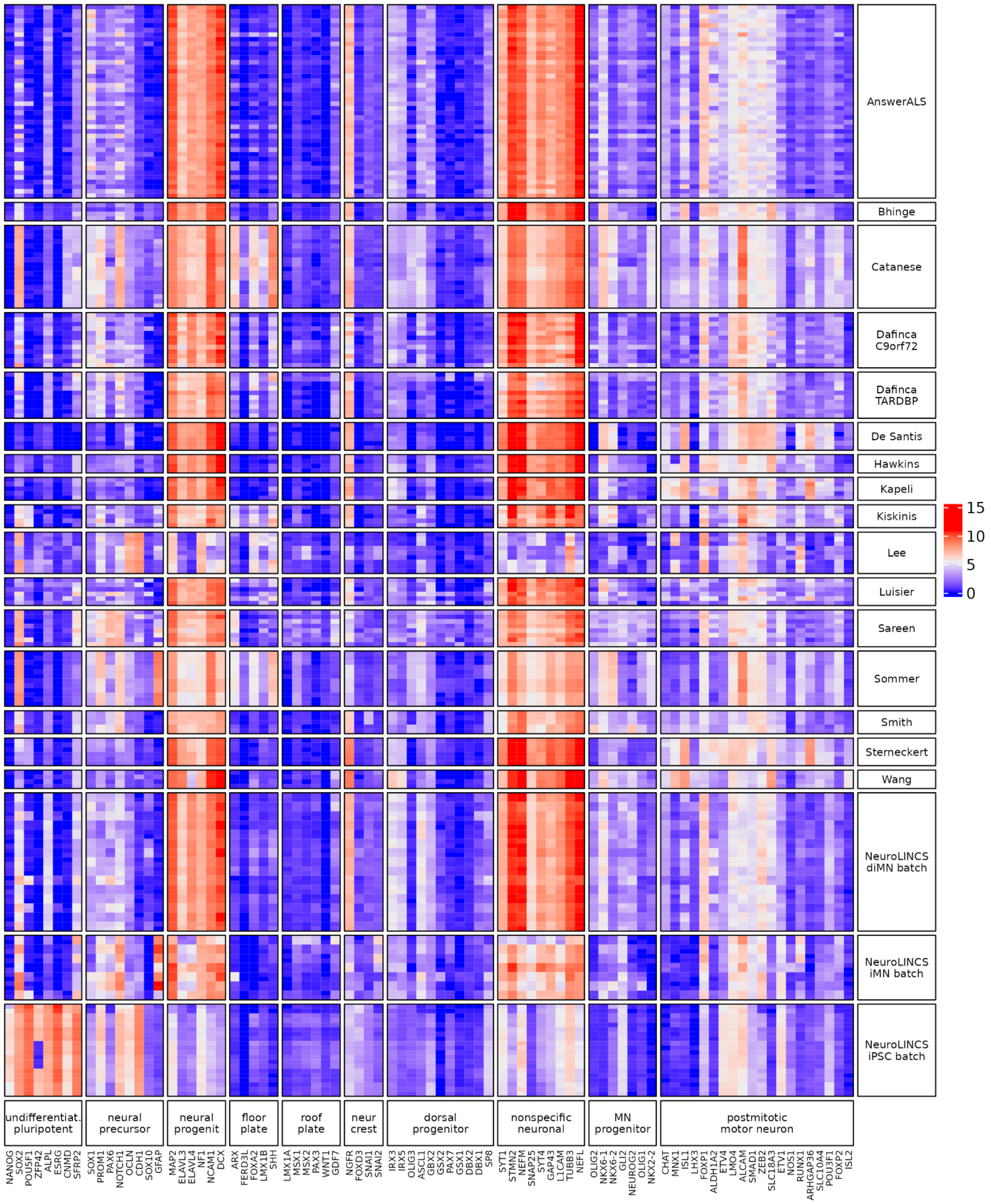

Supplementary Figure 5 Motor neuron differentiation state

Heatmap depicting normalised gene counts of undifferentiated stem cells, neural precursors, neural progenitors, floor plate, roof plate, neural crest and dorsal progenitors, and neuronal markers (columns) across the iPSC-derived spinal motor neuron datasets (rows). Blue represents low gene expression and red represents high expression. To improve the visualisation of all datasets, for AnswerALS only 100 samples are plotted. Lee dataset is removed from the integrated analysis

because of inadequate neuronal marker expression. The NeuroLINCS iPSC batch (bottom block) is not included in the integrated analysis and is only included in this heatmap to enable comparison of iPSMNs with undifferentiated pluripotent iPSCs.

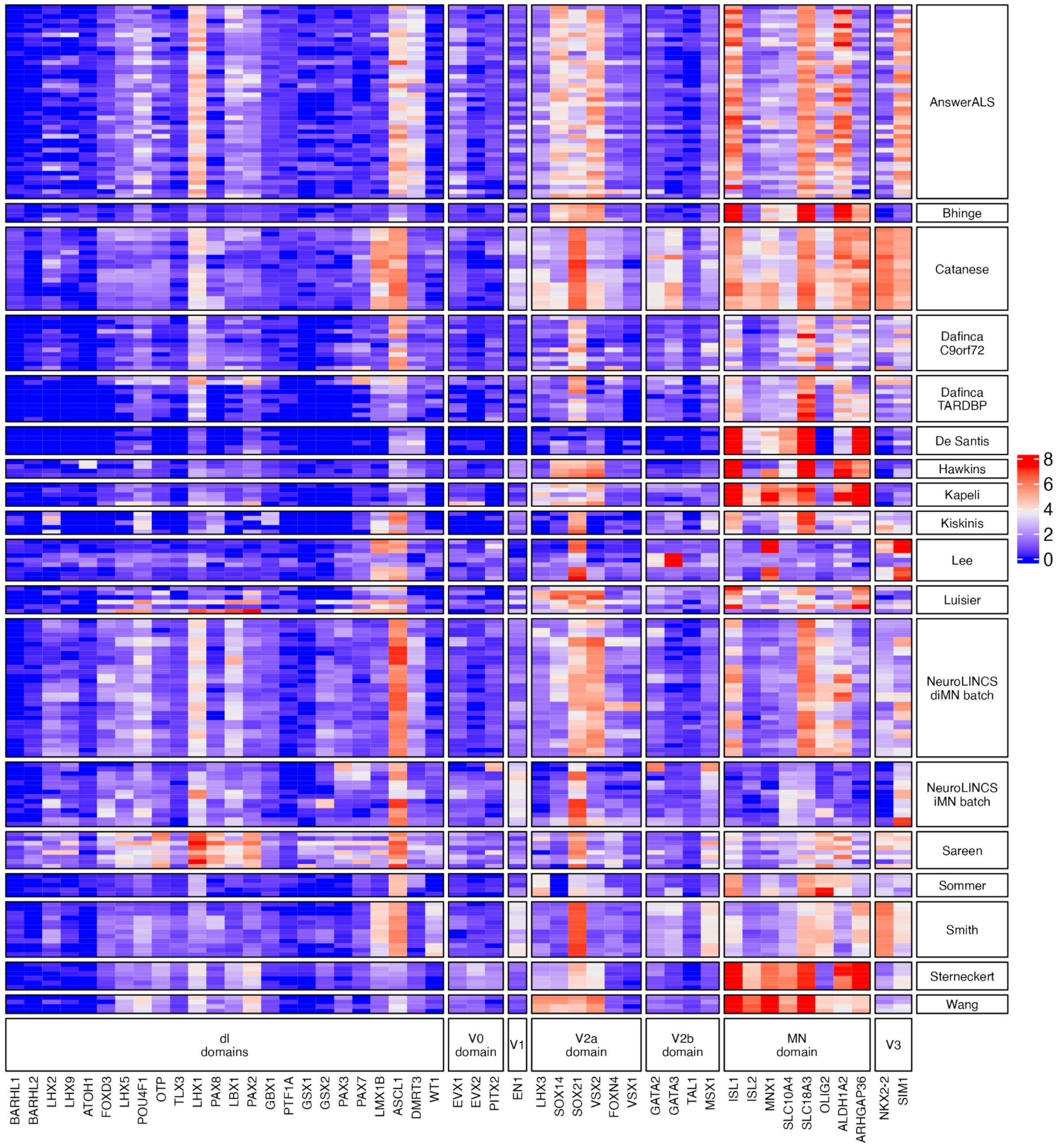

Supplementary Figure 6 Spinal cord dorsoventral identities

Heatmap of normalised gene counts for spinal cord dorsoventral markers from Raynon et al.[16] (columns) across iPSC-derived motor neuron datasets (rows). Blue represents low gene expression and red represents high expression. To improve the visualisation of all datasets, for AnswerALS only 100 samples are plotted.

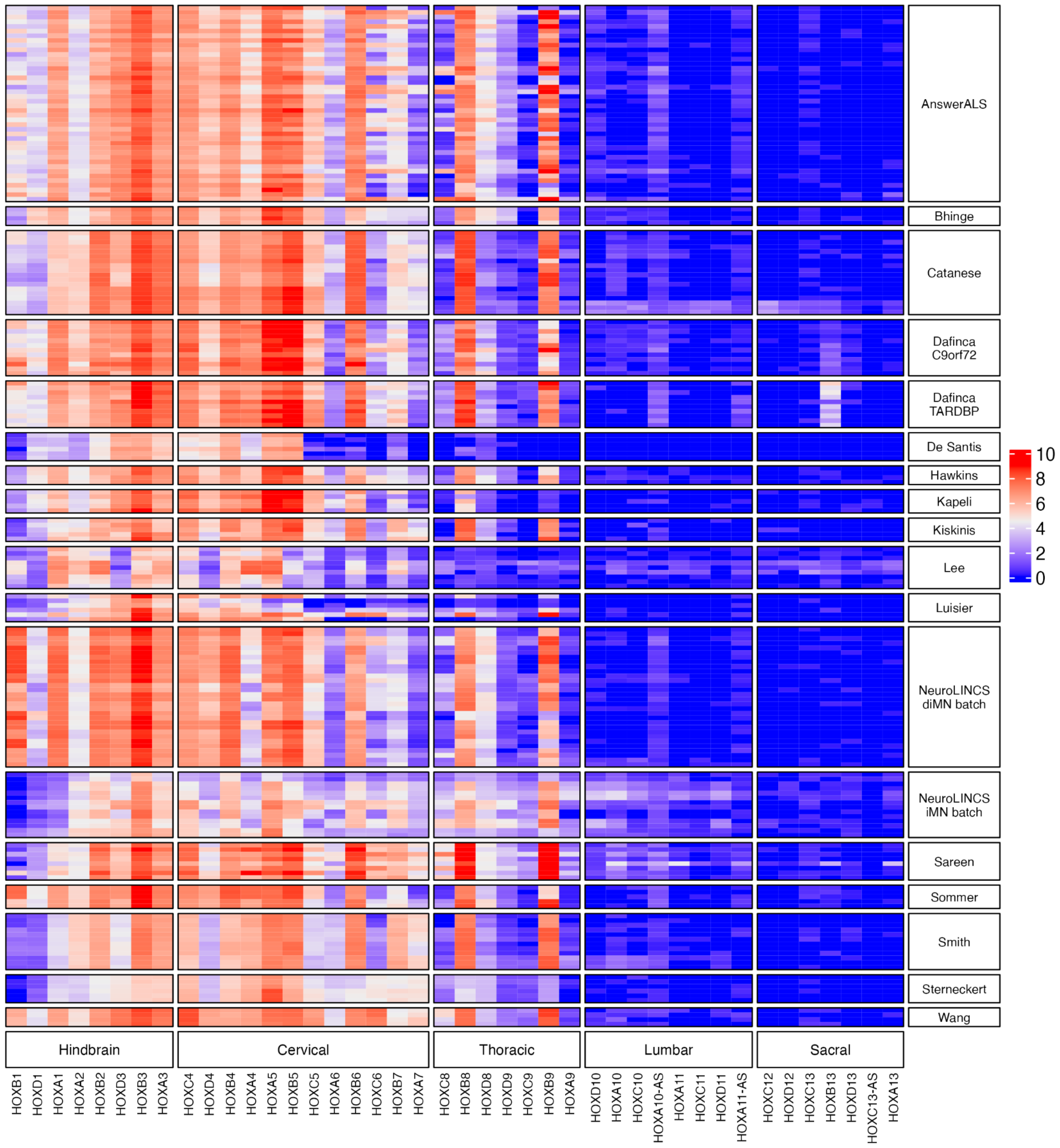

Supplementary Figure 7 HOX marker rostrocaudal regional identities

Heatmap of normalised gene counts for HOX markers (columns) across the iPSC-derived motor neuron datasets (rows). Blue represents low gene expression and red represents high expression. To improve the visualisation of all datasets, for AnswerALS only 100 samples are plotted.

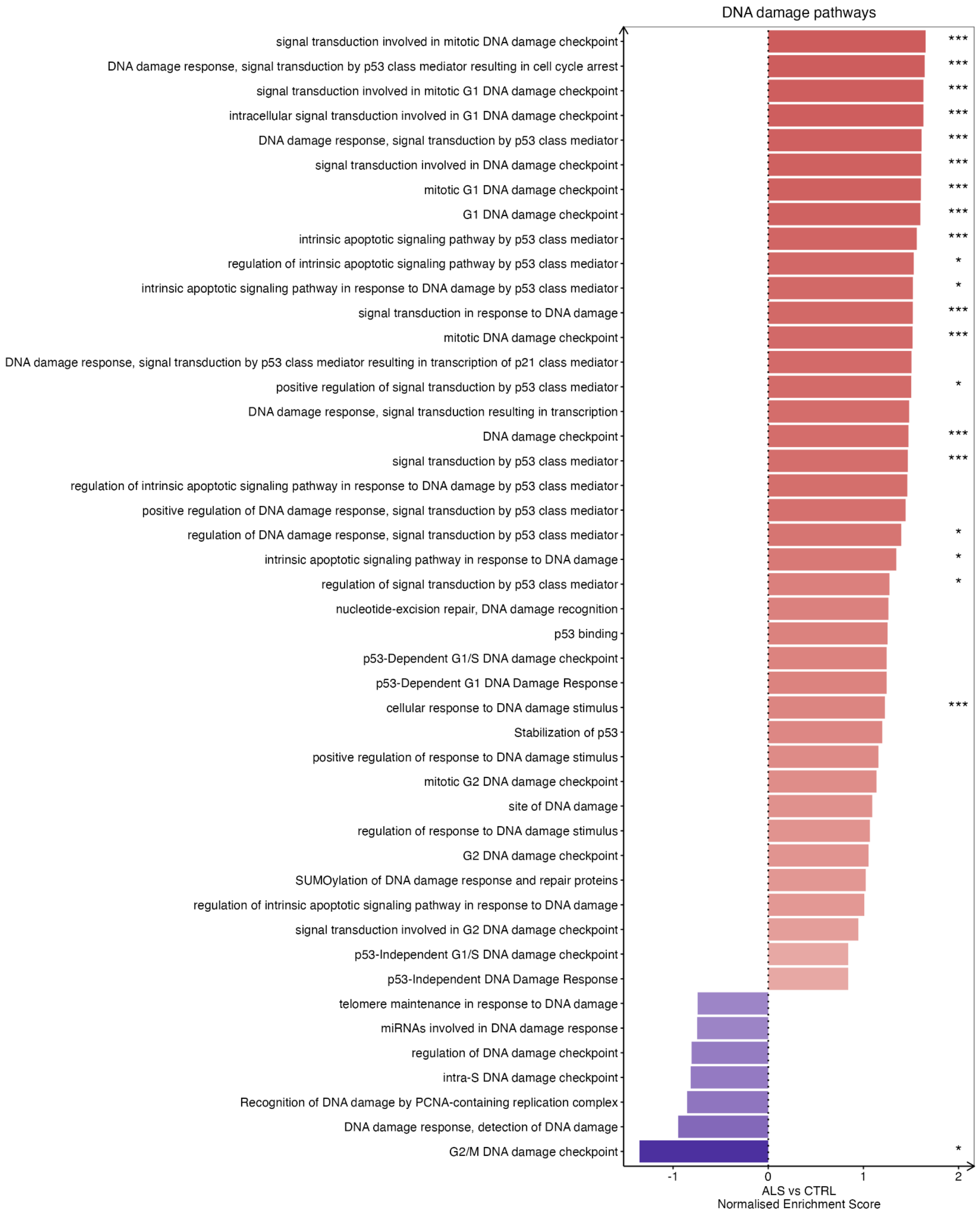

Supplementary Figure 8 Gene set enrichment analysis of DNA damage daughter terms

Daughter terms of DNA damage pathway upregulated in ALS are red (right) and those decreased in ALS iPSMNs are blue (left). Statistics from the hypergeometric test. **** $p < 0.0001$, *** $p < 0.001$, ** $p < 0.01$, * adjusted $p < 0.05$ from GSEA enrichment test.

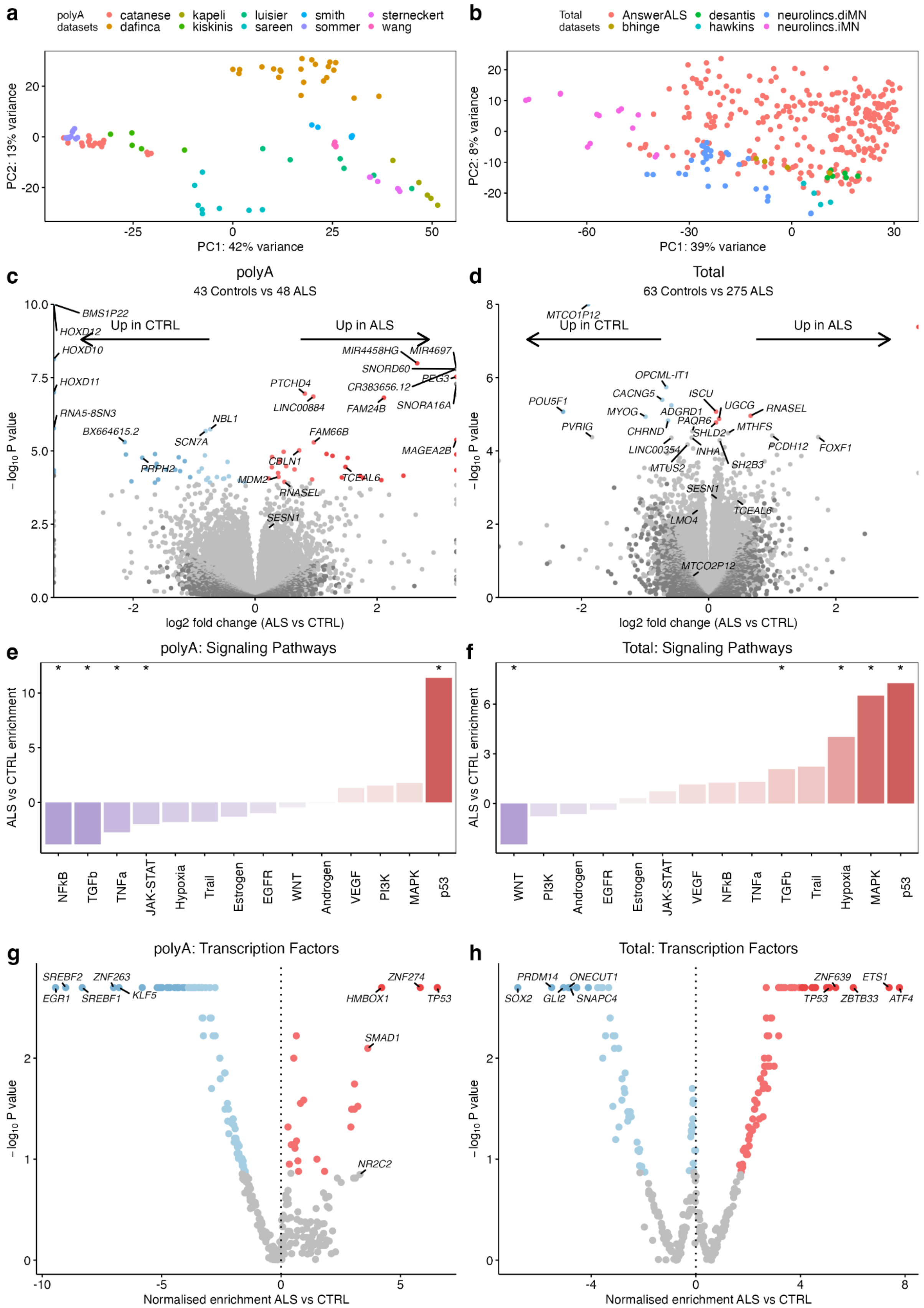

a
polyA datasets
catanese
dafinca
kapeli
kiskinis
luisier
sareen
smith
sommer
sterneckert
wang
PC1: 42% variance
PC2: 13% variance
b
Total datasets
AnswerALS
bhinge
desantis
hawkins
neurolincs.diMN
neurolincs.iMN
PC1: 39% variance
PC2: 8% variance
c
polyA
43 Controls vs 48 ALS
Up in CTRL
Up in ALS
BMS1P22
HOXD12
HOXD10
HOXD11
RNA5-8SN3
BX664615.2
SCN7A
NBL1
PRPH2
MIR4458HG
MIR4697
SNORD60
PEG3
CR383656.12
PTCHD4
LINC00884
FAM24B
SNORA16A
FAM66B
MAGEA2B
CBLN1
MDM2
TCEAL6
RNASEL
SESN1
log2 fold change (ALS vs CTRL)
− log10 P value
d
Total
63 Controls vs 275 ALS
Up in CTRL
Up in ALS
MTCO1P12
OPCML-IT1
CACNG5
ISCU
POU5F1
MYOG
ADGRD1
UGCG
RNASEL
PAQR6
PVRIG
CHRND
SHLD2
MTHFS
PCDH12
FOXF1
LINC00354
INHA
MTUS2
SH2B3
SESN1
TCEAL6
LMO4
MTCO2P12
log2 fold change (ALS vs CTRL)
− log10 P value
e
polyA: Signaling Pathways
ALS vs CTRL enrichment
NFkB
TGFb
TNFa
JAK-STAT
Hypoxia
Trail
Estrogen
EGFR
WNT
Androgen
VEGF
PI3K
MAPK
p53
f
Total: Signaling Pathways
ALS vs CTRL enrichment
WNT
PI3K
Androgen
EGFR
Estrogen
JAK-STAT
VEGF
NFkB
TNFa
TGFb
Trail
Hypoxia
MAPK
p53
g
polyA: Transcription Factors
SREBF2
ZNF263
EGR1
SREBF1
KLF5
ZNF274
HMBOX1
TP53
SMAD1
NR2C2
Normalised enrichment ALS vs CTRL
− log10 P value
h
Total: Transcription Factors
PRDM14
ONECUT1
SOX2
GLI2
SNAPC4
ZNF639
ETS1
TP53
ZBTB33
ATF4
Normalised enrichment ALS vs CTRL
− log10 P value

Supplementary Figure 9 Sensitivity analysis of pan ALS iPSMNs in poly(A) and total RNA samples separately

a-b: Principle component analysis plots of normalised transformed gene expression from polyA (a) and total (b) iPSMN samples separately, coloured by dataset.

c-d: Volcano plots showing log_2 fold change in differential gene expression in pan-ALS compared to control iPSMNs in polyA (c) and total (d) RNA library preparation samples from the Wald test. Significantly changed (FDR < 0.05) genes in ALS are coloured red (increased) and blue (decreased).

e-f: Signalling pathway activities from PROGENy showing ALS versus control iPSMN normalised enrichment scores (y-axis) in polyA (e) and total (f) samples. Pathways that are increased in ALS are coloured red whilst pathways decreased are coloured blue. Statistics from the weighted mean method. * represents enrichment test p-value < 0.05

g-h: Transcription factor activities inferred from their respective regulon gene expression changes in ALS versus control iPSMNs using DoRothEA in polyA (g) and total (h) samples. Normalised enrichment in ALS versus control (x-axis) is plotted according to the DoRothEA p-value from the enrichment test (y-axis).

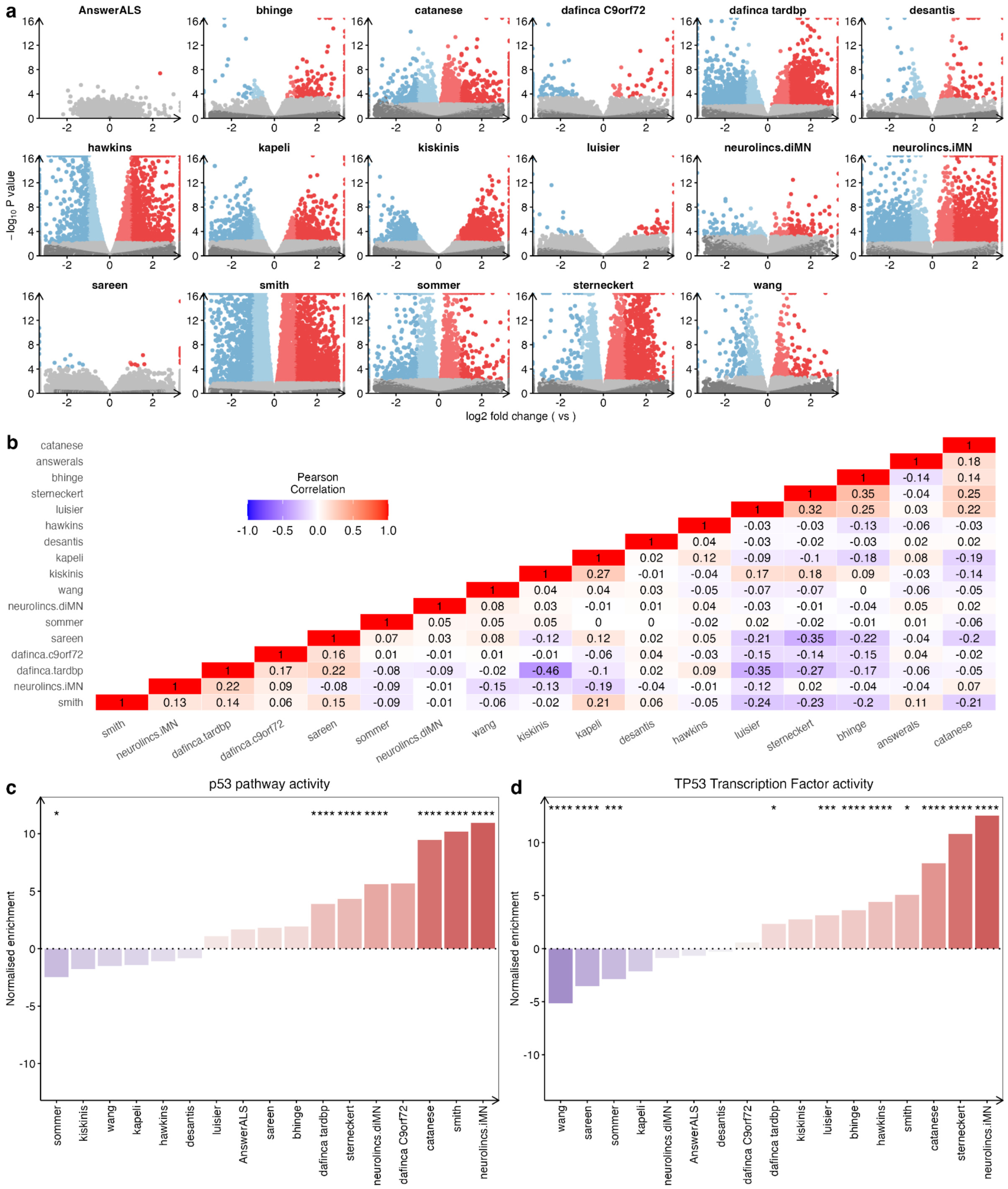

Supplementary Figure 10 Analysis of each iPSMN dataset separately

a: Volcano plots showing log_2 fold change in differential gene expression in ALS compared to control iPSMNs in each dataset from the Wald test. Significantly changed (FDR < 0.05) genes in ALS are coloured red (increased) and blue (decreased).

b: Heatmap showing the Pearson's correlation coefficient for transcriptome-wide changes between each dataset.

c p53 signalling pathway activity showing ALS versus control iPSMNs normalised enrichment scores

(y-axis) in each dataset from the weighted mean method. Pathways that are increased in ALS are coloured red whilst pathways decreased are coloured blue.

d: TP53 transcription factor activity in ALS versus control iPSMNs in each dataset. Normalised enrichment in ALS versus control (x-axis) is plotted for each dataset (y-axis). * represents enrichment test p-value < 0.05, ** p < 0.01, *** p < 0.001, **** p < 0.0001.

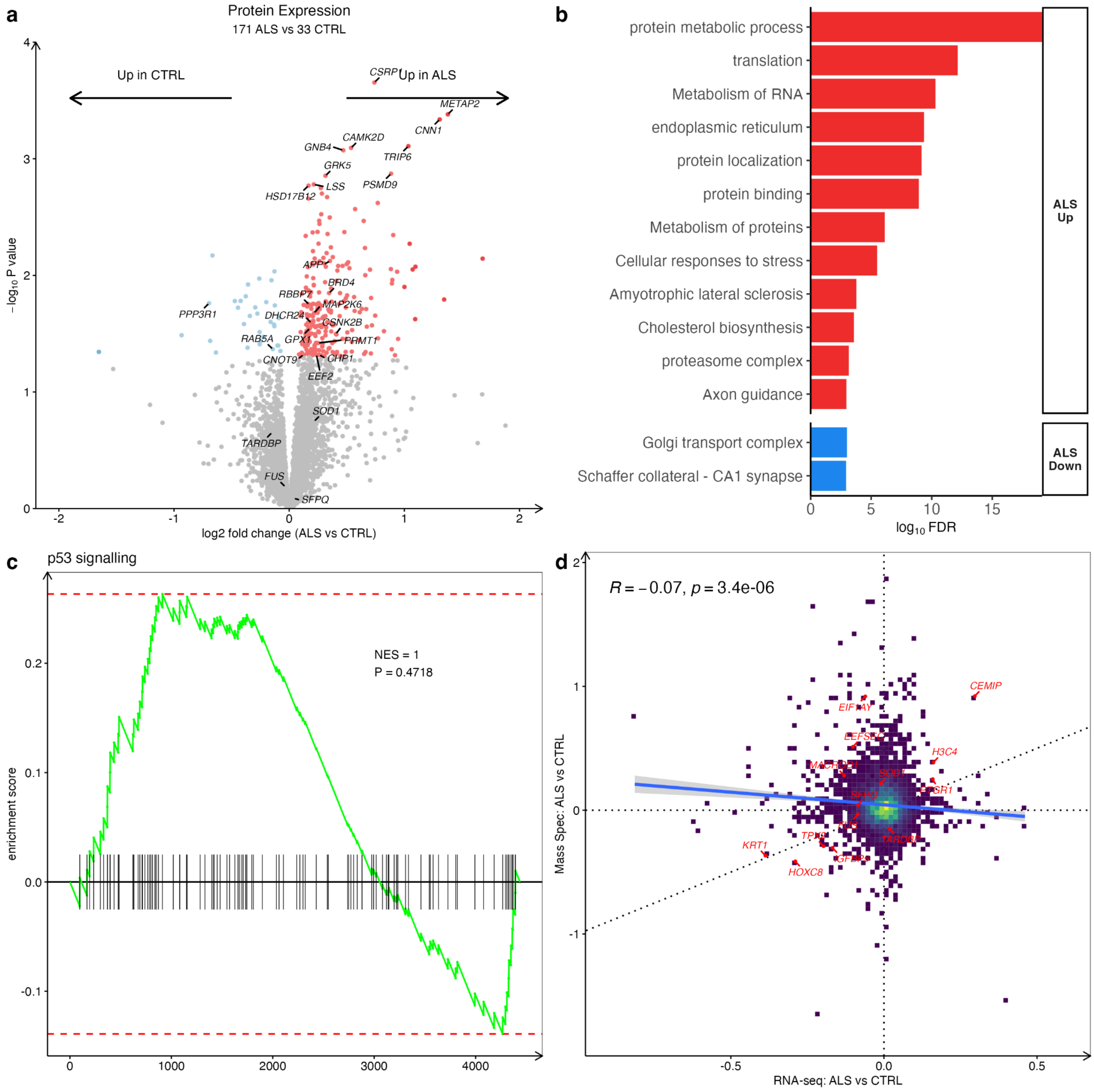

Supplementary Figure 11 Mass spectrometry of iPSMNs in AnswerALS

a: Volcano plot showing differential protein expression changes in ALS versus control iPSMNs from Answer ALS mass spectrometry data using the Wald test. There were no significant proteins at FDR < 0.05 and a more lenient threshold of P value < 0.05 is used to colour significantly changed genes.

b: Gene Ontology terms enriched in up-regulated (red) and down-regulated (blue) differentially

expressed proteins in ALS versus control iPSMNs. P-values are from the hypergeometric test.

c: Protein set enrichment analysis of signal transduction by p53 (GO:0072331, n = 103) in ALS versus control using the permutation test. NES, normalised enrichment score.

d: Scatterplot of gene expression changes (Wald test statistic; x-axis) against protein expression changes (y-axis) in ALS versus control iPSMNs. Overlapping differentially expressed genes/proteins are coloured red. The solid blue line represents the linear correlation and Pearson correlation.

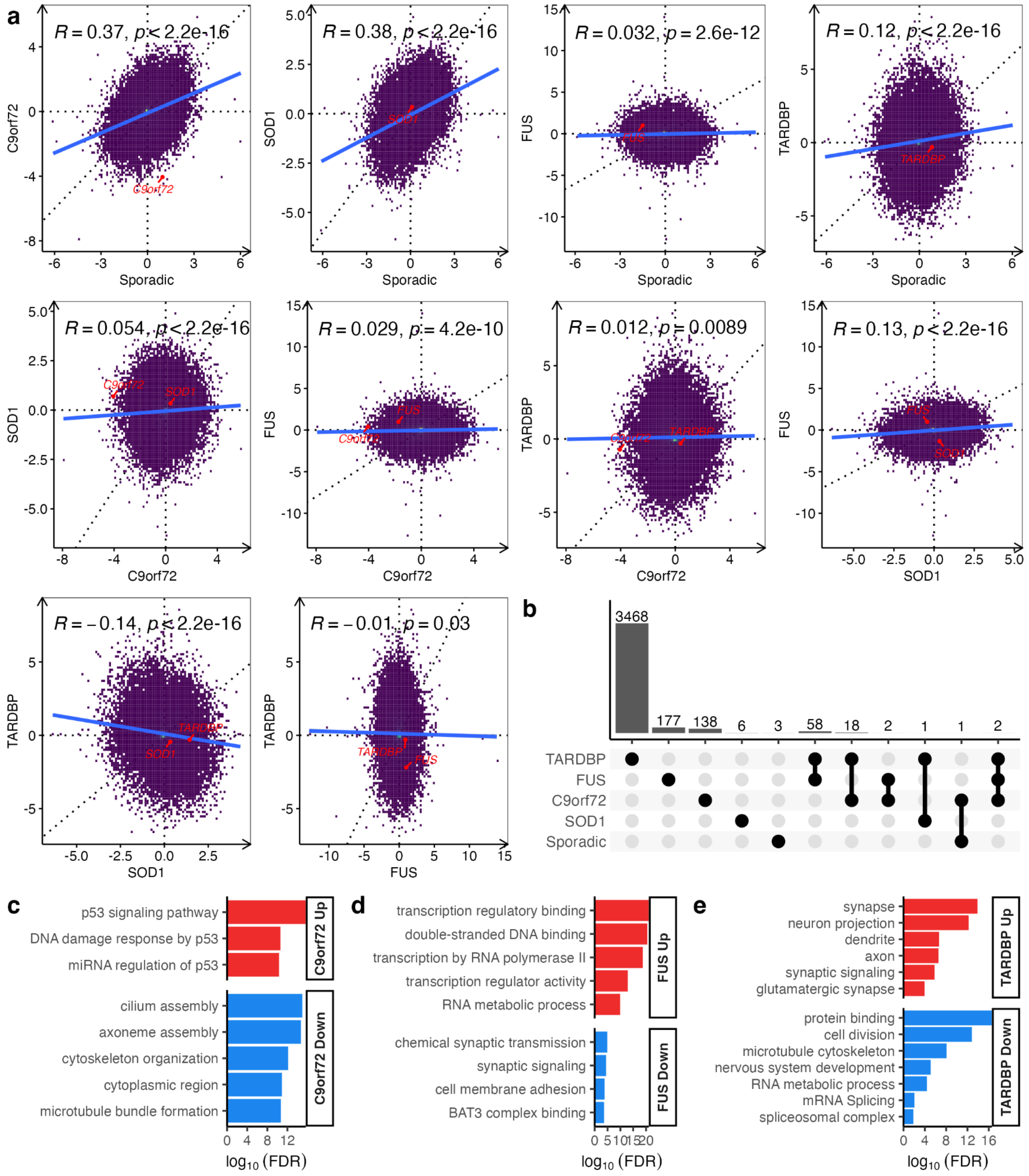

Supplementary Figure 12 Correlating iPSMN gene expression changes between genetic backgrounds

a, Wald test statistics represent the sign and magnitude of the differential gene expression test for each gene compared between each mutation pair. The Pearson correlation value is at the top of each plot.

b, Upset plot showing overlapping differentially expressed genes (FDR < 0.05) between each genetic background.

c-e, Functional enrichment terms enriched in (c) C9orf72, (d) FUS and (e) TARDBP using the hypergeometric test. Upregulated terms are coloured red and downregulated are blue. There were no terms enriched in SOD1 or sporadic genetic backgrounds.

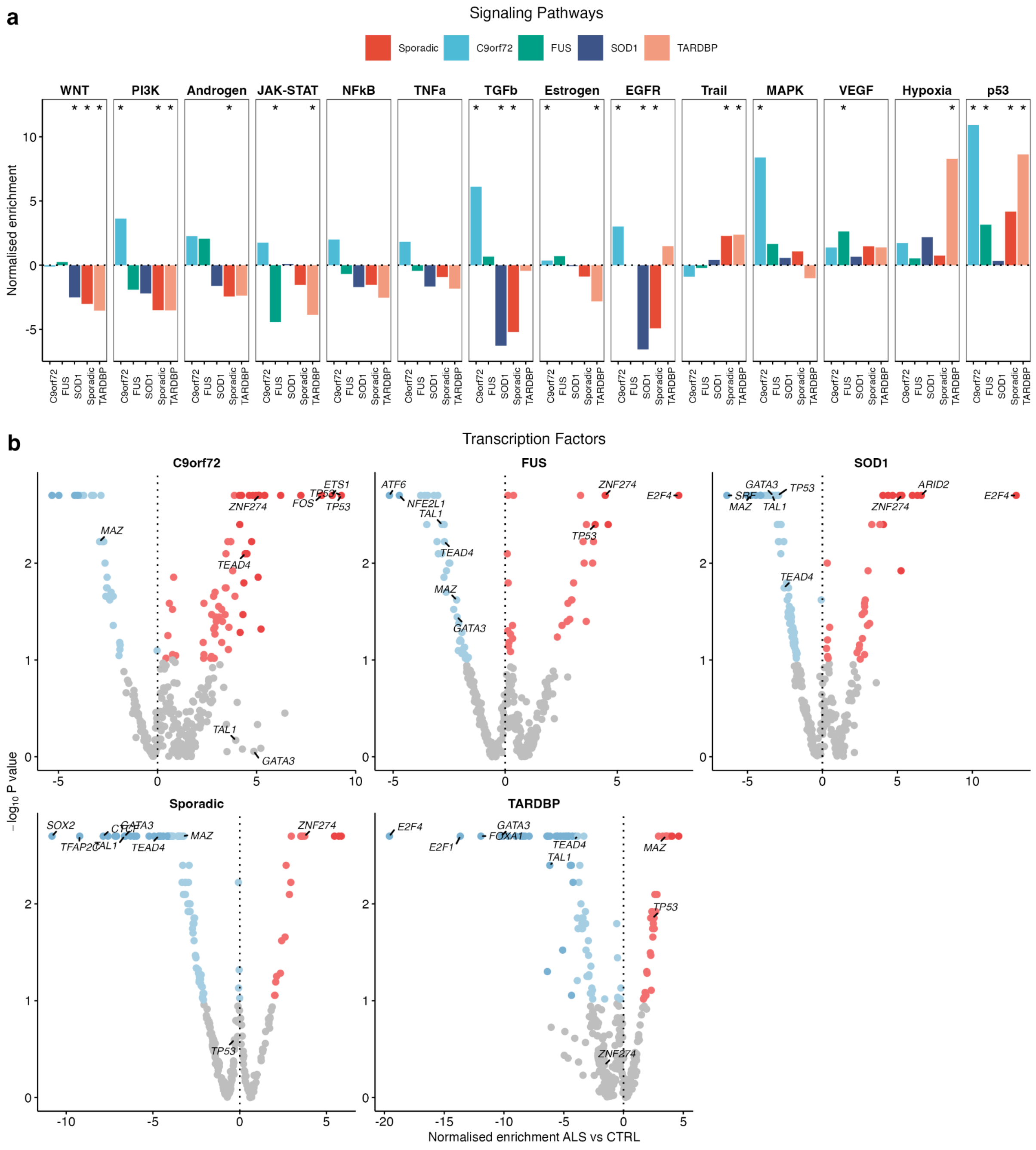

Supplementary Figure 13 iPSMN signalling pathway and transcription factor activities between genetic backgrounds

Normalised enrichment scores in the distinct ALS genetic backgrounds versus controls for (a) PROGENy signalling pathways and (b) DoRothEA transcription factor activities. Pathways that are increased in ALS are coloured red whilst pathways decreased are coloured blue. Statistics are from the weighted mean method. * represents enrichment test p-value < 0.05

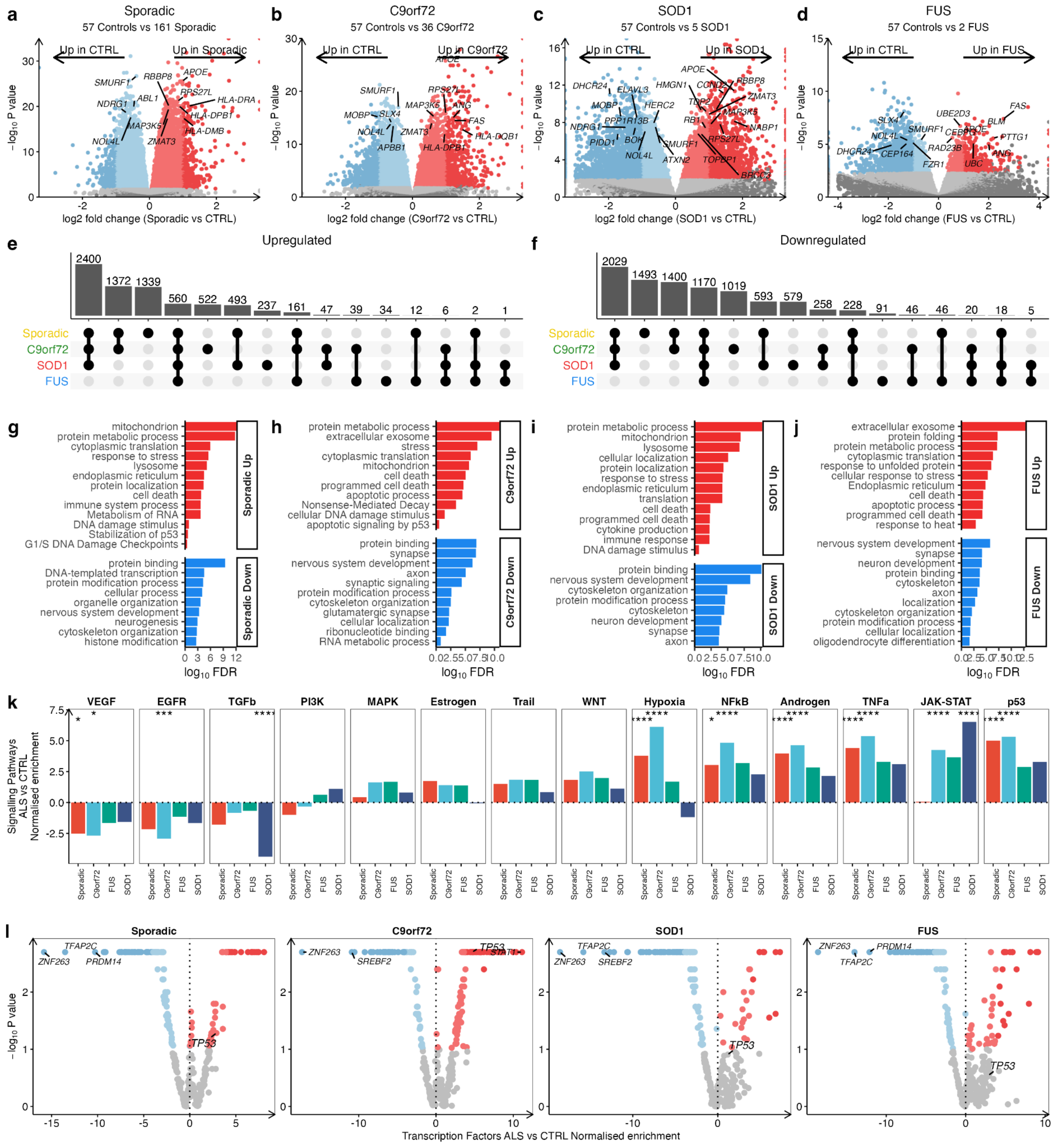

Supplementary Figure 14 Postmortem spinal cord gene expression changes between genetic backgrounds

a-d, Volcano plots showing differential gene expression comparing ALS to control post-mortem tissue in each ALS genetic background from the Wald test. Genes coloured red are significantly increased in the ALS subgroup, and genes coloured blue are decreased in the ALS subgroup. **e-f,** Upset plots showing overlapping (j) upregulated and (k) downregulated differentially expressed genes (FDR < 0.05) between each genetic subgroup. **g-j,** Functional enrichment terms enriched in (e) Sporadic, (f) C9orf72, (g) SOD1, and (h) FUS using the hypergeometric test. Upregulated terms are coloured red and downregulated are blue. **k-l,** normalised enrichment scores in the distinct ALS genetic subgroups

versus controls for (l) PROGENy signalling pathways and (m) DoRothEA transcription factor activities using the weighted mean method.

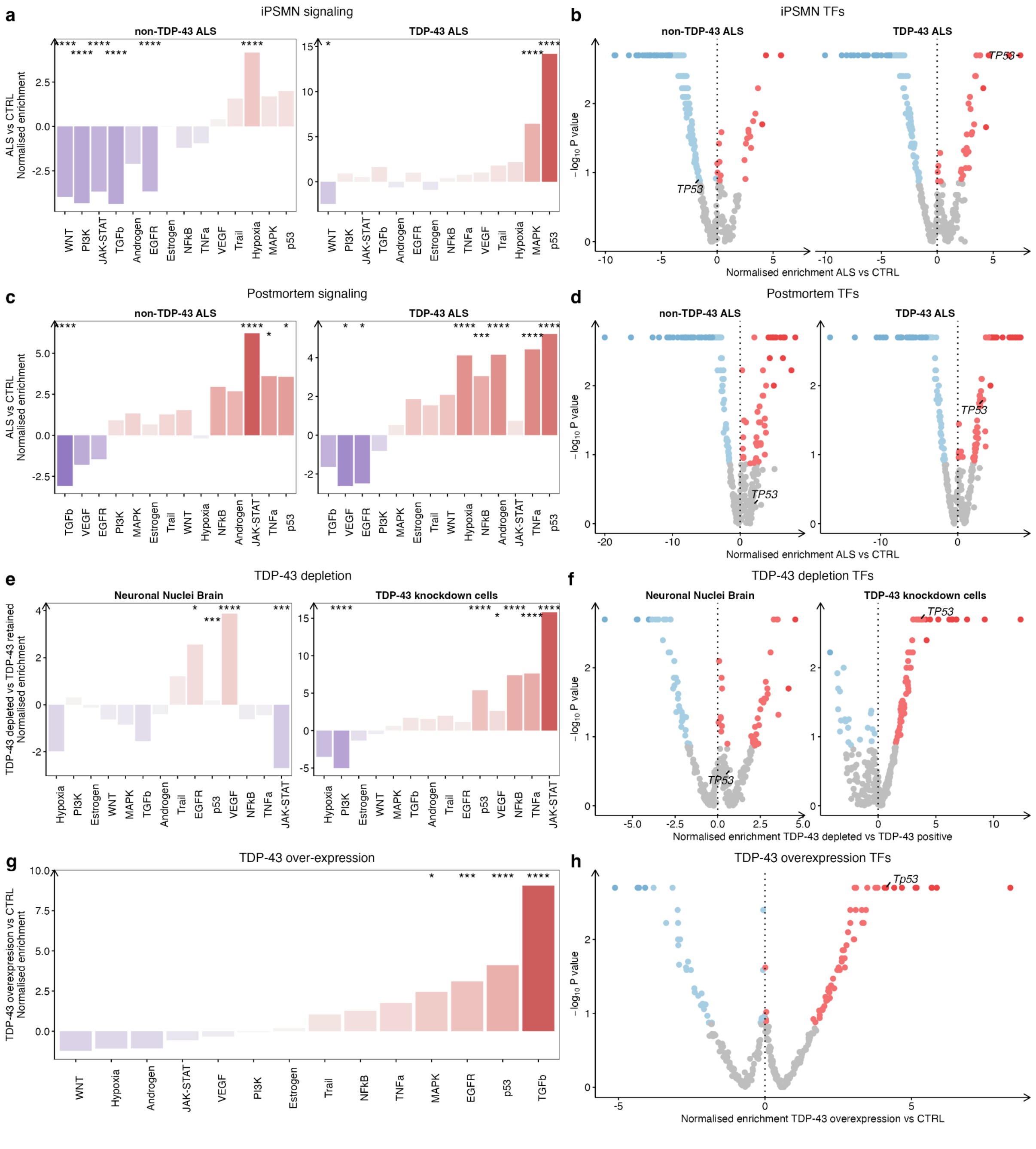

Supplementary Figure 15 TDP-43 loss of function contributes to p53 signaling activation

PROGENy signalling pathway barcharts (left) and DoRothEA transcription factor activities volcano plot (right) in (a-b) non-TDP-43 proteinopathy ALS (i.e. SOD1 and FUS mutant) and TDP-43 proteinopathy ALS iPSMNs; (c-d) non-TDP-43 ALS (i.e. SOD1 and FUS mutant) and TDP-43 ALS post-mortem; (e-f) FACS sorted neuronal nuclei depleted of TDP-43 (n = 14) and TDP-43 knockdown

cell models (n = 49); (g-h) TDP-43 overexpressing mouse neurons. Statistics are from the weighted mean method with p-values from the enrichment test.

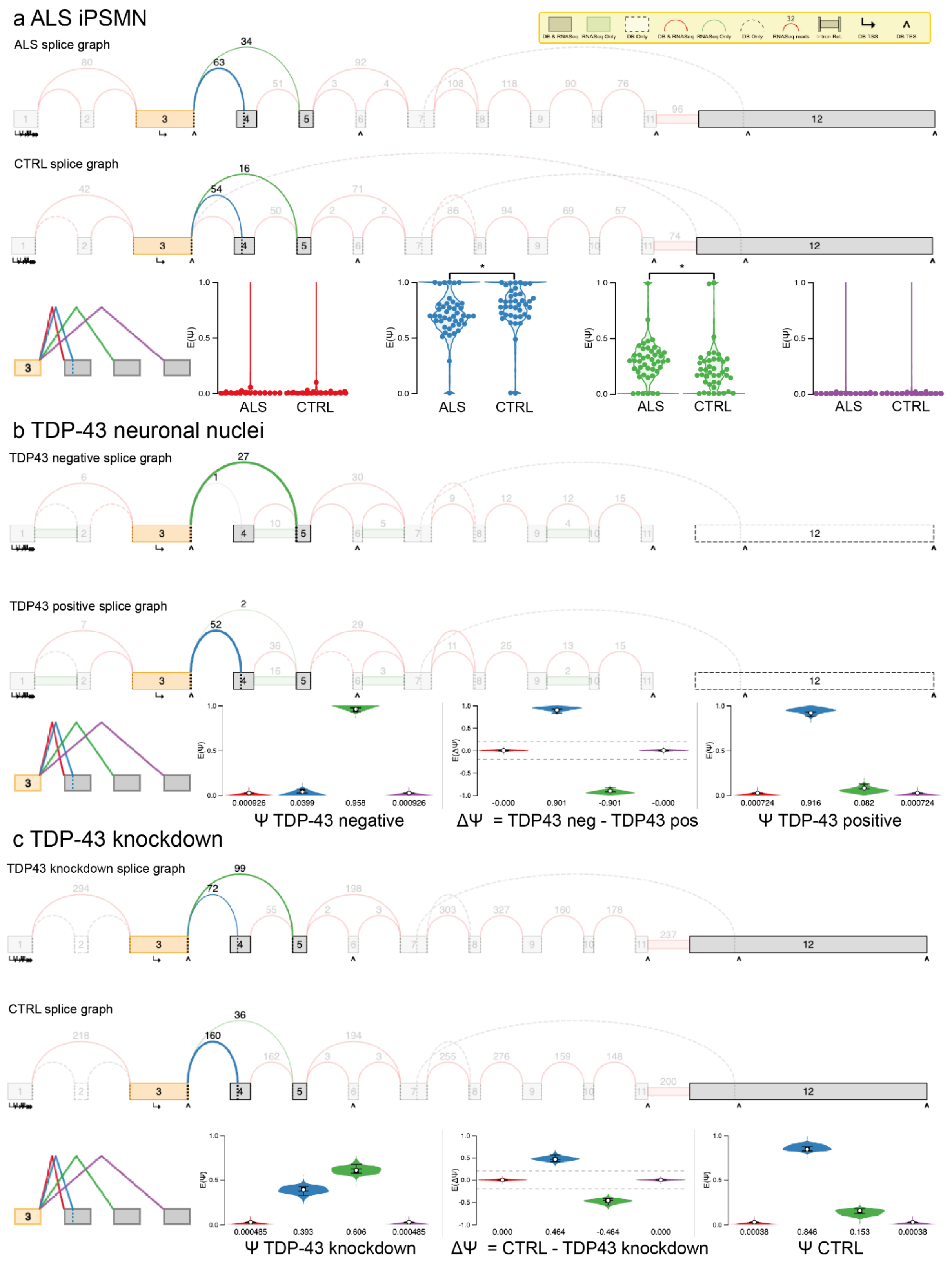

Supplementary Figure 16 POLDIP3 splicing event in ALS iPSMNs, TDP-43 depleted neuronal nuclei and TDP43 knockdown iNeurons

MAJIQ voila view of POLDIP3 multi-exon skipping event (coordinates start 42,602,768 and end 42,603,160) in (a) ALS (n = 48) versus control (n = 43) iPSMNs (using heterogen function), (b) TDP-43 positive (n = 7) versus TDP-43 negative (n = 7) FACS sorted neuronal nuclei from Liu et al 2019, and (c) TDP-43 knockdown (n = 3) versus control (n = 4) iNeurons from Leigh-Brown et al 2022 (using deltaPSI function). In the splice graphs, the green exon skipping event (coordinates 42,602,056-42,602,770) is increased in ALS iPSMN, TDP-43 neuronal nuclei depletion, and TDP-43 knockdown compared to their respective controls. Conversely, the blue exon skipping event is decreased in ALS iPSMNs, TDP-43 neuronal nuclei depletion, and TDP-43 knockdown compared to their respective controls. Statistics are from the TNOM test.

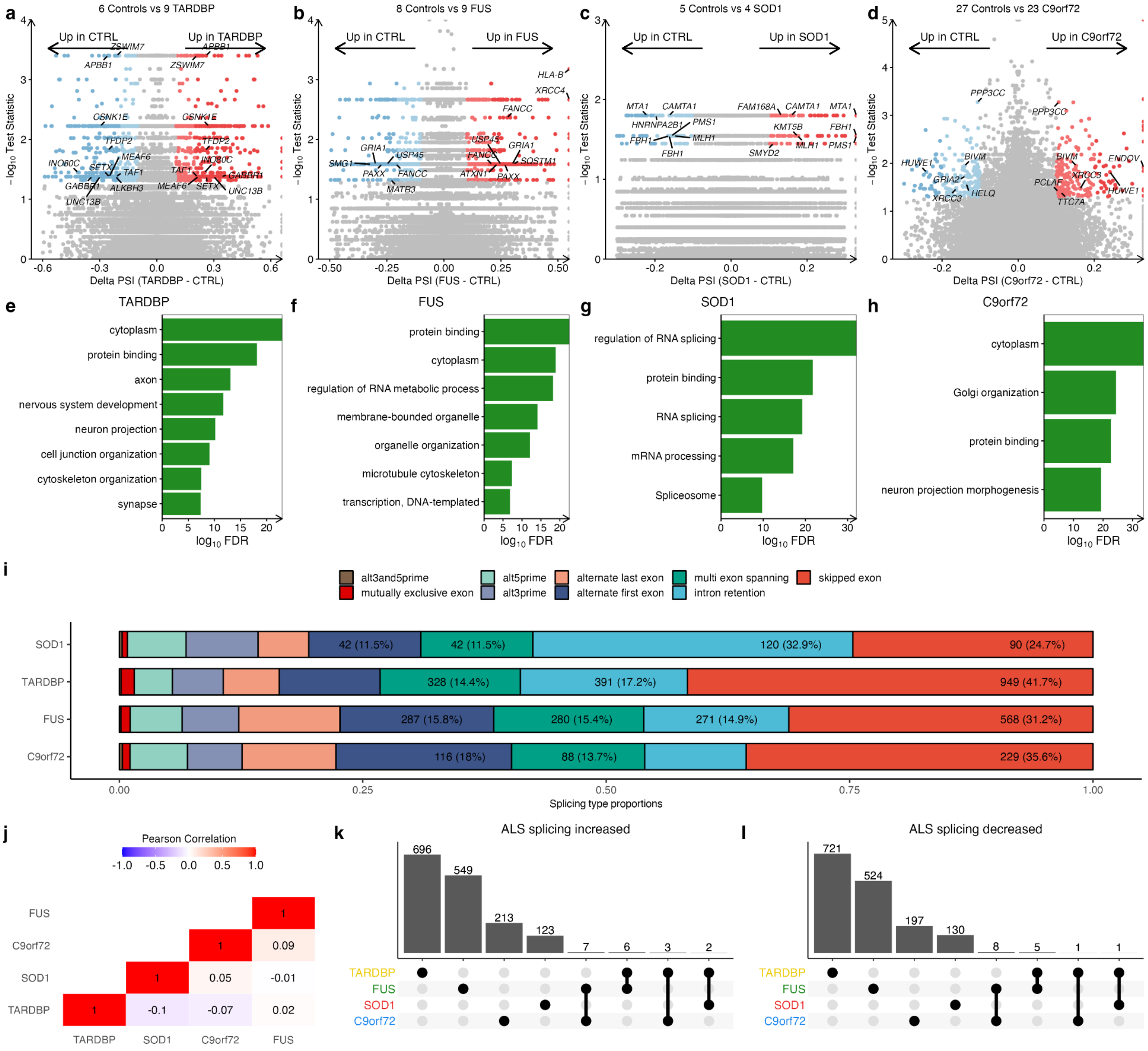

Supplementary Figure 17 Splicing changes in distinct ALS genetic backgrounds

a-d: Volcano plots showing splicing changes in ALS versus control iPSMNs (delta PSI, x-axis) against $-\log_{10}$ TNOM test statistic for (a) *TARDBP* mutant, (b) *FUS* mutant, (c) *SOD1* mutant, and (d) *C9orf72* mutant iPSMNs. Splice events significantly increased in ALS are coloured red, and those significantly decreased are coloured blue.

e-h: Functionally enriched terms amongst differential splice events in (e) *TARDBP* mutant, (f) *FUS* mutant, (g) *SOD1* mutant, and (h) *C9orf72* mutant iPSMNs from the hypergeometric test.

i: Barchart showing proportions of each splicing type in significant splice events in *SOD1, TARDBP, FUS,* and *C9orf72* mutant iPSMNs. Labels depict the numbers and percent of splice events for the most common splicing types.

j: Heatmap showing the Pearson's correlation coefficient for transcriptome-wide splicing changes between each mutant group.

k-l: UpSet plots showing the numbers of overlapping splice events (k) increased and (l) decreased between mutations.

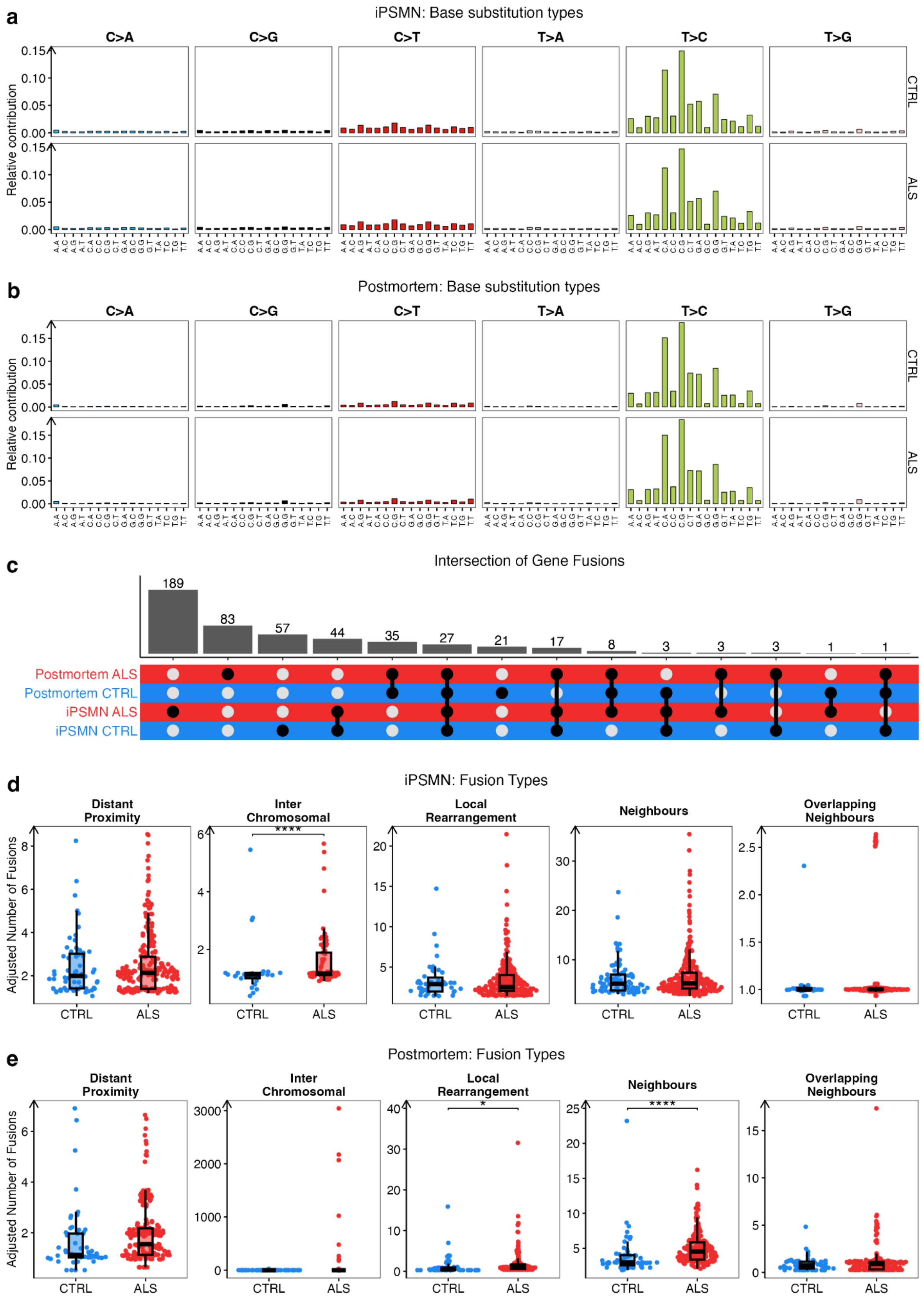
a
iPSMN: Base substitution types
C>A
C>G
C>T
T>A
T>C
T>G
Relative contribution
0.15
0.10
0.05
0.00
CTRL
ALS
b
Postmortem: Base substitution types
C>A
C>G
C>T
T>A
T>C
T>G
Relative contribution
0.15
0.10
0.05
0.00
CTRL
ALS
c
Intersection of Gene Fusions
189
83
57
44
35
27
21
17
8
3
3
3
1
1
Postmortem ALS
Postmortem CTRL
iPSMN ALS
iPSMN CTRL
d
iPSMN: Fusion Types
Distant Proximity
Inter Chromosomal
Local Rearrangement
Neighbours
Overlapping Neighbours
Adjusted Number of Fusions
****
CTRL
ALS
e
Postmortem: Fusion Types
Distant Proximity
Inter Chromosomal
Local Rearrangement
Neighbours
Overlapping Neighbours
Adjusted Number of Fusions
*
****
CTRL
ALS

Supplementary Figure 18 SNV and fusion types in genetic subgroups

a-b, Relative contribution of each base substitution type (96 trinucleotide mutation profile) in CTRL (top) and ALS (bottom) (a) iPSMNs and (b) postmortem tissue.

c, UpSet plot depicting overlapping unique gene fusions in iPSMNs and postmortem ALS and CTRL samples.

d-e, Adjusted numbers of each type of gene fusion event per sample in ALS (red) and CTRL (blue) samples in (d) iPSMNs (ALS n = 306; CTRL n = 90) and (e) postmortem (ALS n = 214; CTRL n = 57). A generalised linear model with Poisson distribution was fit to compare ALS with control, adjusting for coverage, age, and dataset. Plotted values represent the partial residuals after adjusting for dataset batches, read depth and age and statistics are from the generalised linear model Wald test accounting for dataset batches, age, and read coverage. **** $p < 0.0001$, *** $p < 0.001$, ** $p < 0.01$, * $p < 0.05$. In the boxplots, whiskers (error bars) represent 1.5 times the interquartile range, the hinges correspond to the first and third quartiles, and the centre represents the median.
