# Supplementary material for: Integrated transcriptome landscape of ALS identifies genome instability linked to TDP-43 pathology
Source: Nat Commun. 2023 Apr 20;14:2176. doi: 10.1038/s41467-023-37630-6 (PMC10119258; doi:10.1038/s41467-023-37630-6)
Supplement: Supplementary file 3 — Description of Additional Supplementary Files [file 41467_2023_37630_MOESM3_ESM.pdf]

File Name: Supplementary Data 1
Description: iPSMN differentiation protocols and RNA-seq library strategies

File Name: Supplementary Data 2
Description: iPSMN RNA-seq quality control metrics

File Name: Supplementary Data 3
Description: Differential gene expression results from pan ALS versus control iPSMNs

File Name: Supplementary Data 4
Description: Transcription factor activities inferred with Dorothea in ALS versus control iPSMNs

File Name: Supplementary Data 5
Description: Differential protein expression results from pan ALS versus control iPSMNs

File Name: Supplementary Data 6
Description: Differential gene expression in each ALS genetic background in iPSMNs

File Name: Supplementary Data 7
Description: Overlapping differentially expressed genes in ALS versus control between iPSMNs and post-mortem

File Name: Supplementary Data 8
Description: TDP43 knock down dataset details

File Name: Supplementary Data 9
Description: Differential splicing events in ALS versus control iPSMNs

File Name: Supplementary Data 10
Description: Differential splicing events in in each ALS genetic background in iPSMNs

File Name: Supplementary Data 11
Description: Differential splicing events in ALS versus control post-mortem tissue

File Name: Supplementary Data 12
Description: Numbers of somatic mutations in iPSMNs

File Name: Supplementary Data 13
Description: Numbers of somatic mutations in post-mortem tissue

File Name: Supplementary Data 14
Description: Numbers of gene fusions in iPSMNs and post-mortem tissue
