# Supplementary material for: Integrated transcriptome landscape of ALS identifies genome instability linked to TDP-43 pathology
Source: Nat Commun. 2023 Apr 20;14:2176. doi: 10.1038/s41467-023-37630-6 (PMC10119258; doi:10.1038/s41467-023-37630-6)
Supplement: Supplementary file 5 — Reporting Summary [file 41467_2023_37630_MOESM5_ESM.pdf]

Corresponding author(s): Oliver Ziff, Rickie Patani

Last updated by author(s): Feb 24, 2023

Reporting Summary

Nature Portfolio wishes to improve the reproducibility of the work that we publish. This form provides structure for consistency and transparency in reporting. For further information on Nature Portfolio policies, see our Editorial Policies and the Editorial Policy Checklist.

Statistics

For all statistical analyses, confirm that the following items are present in the figure legend, table legend, main text, or Methods section.

n/a	Confirmed	
[ ]	[x]	The exact sample size (*n*) for each experimental group/condition, given as a discrete number and unit of measurement
[ ]	[x]	A statement on whether measurements were taken from distinct samples or whether the same sample was measured repeatedly
[ ]	[x]	The statistical test(s) used AND whether they are one- or two-sided<br>*Only common tests should be described solely by name; describe more complex techniques in the Methods section.*
[ ]	[x]	A description of all covariates tested
[ ]	[x]	A description of any assumptions or corrections, such as tests of normality and adjustment for multiple comparisons
[ ]	[x]	A full description of the statistical parameters including central tendency (e.g. means) or other basic estimates (e.g. regression coefficient) AND variation (e.g. standard deviation) or associated estimates of uncertainty (e.g. confidence intervals)
[ ]	[x]	For null hypothesis testing, the test statistic (e.g. F, t, r) with confidence intervals, effect sizes, degrees of freedom and P value noted<br>*Give P values as exact values whenever suitable.*
[x]	[ ]	For Bayesian analysis, information on the choice of priors and Markov chain Monte Carlo settings
[x]	[ ]	For hierarchical and complex designs, identification of the appropriate level for tests and full reporting of outcomes
[ ]	[x]	Estimates of effect sizes (e.g. Cohen's *d*, Pearson's *r*), indicating how they were calculated

Our web collection on statistics for biologists contains articles on many of the points above.

Software and code

Policy information about availability of computer code

Data collection

All software is freely available to the public and accessible via github. The following links provide unrestricted access:

The main github repository containing the full code to reproduce the figures, the results and supplementary tables: https://github.com/ojziff/als_genome_instability

Supporting repositories:
nf-core https://nf-co.re/ is a repository of gold-standard pipelines built using Nextflow for reproducible analysis of sequencing data. The following nfcore pipelines were used in this study:
nfcore/fetchngs: https://nf-co.re/fetchngs to download sequencing data from public repositories
nfcore/rnaseq: https://nf-co.re/rnaseq to perform quality control and process raw fastq files
nfcore/rnavar: https://nf-co.re/rnavar to identify variants in the fastq files covered in Figure 5
nfcore/rnafusion: https://nf-co.re/rnafusion to identify gene fusions in the fastq files covered in Figure 5
MutationalPatterns R package was used to characterise genomic variants: https://bioconductor.org/packages/release/bioc/html/MutationalPatterns.html covered in Figure 5
DecoupleR package infers signalling pathway (PROGENy) and transcription factor (DoRothEA) activities from gene expression data: https://www.bioconductor.org/packages/release/bioc/html/decoupleR.html
MAJIQ is a tool used to analyse alternative splicing from RNA sequencing data covered in Figure 4: https://majiq.biociphers.org/

For the majority of analyses the statistical software R (v4.1.3) was used. The following packages were used:
tidyverse suite of packages 1.3.1 (tidyr 1.2.0, tibble 3.1.7, ggplot2 3.3.6, ggupset 0.3.0, ggforce 0.3.3, ggsci 2.9, ggpubr 0.4.0, ggplotify 0.1.0,

ggvenn 0.1.9)
janitor 2.1.0
ComplexHeatmap 2.14
biomaRt 2.50.3
limma 3.50.3
GenomicRanges 1.46.1
DESeq2 1.34.0
data.table 1.14.2
gprofiler2 0.2.1
clusterProfiler 4.2.2
patchwork 1.1.1
fgsea 1.20.0
decoupleR 2.0.1
MutationalPatterns 3.4.1

Data analysis

Gene expression
Differential gene expression comparisons were performed with DESeq2 v1.34.0 on Salmon quantified transcript counts.
Gene enrichment analysis was performed with gprofiler2 v0.2.1
Gene Set Enrichment Analysis (GSEA) was performed with fgsea v1.20.0
DoRoTHEA & PROGENy were performed with DecoupleR v 2.0.1

Alternative splicing
MAJIQ v2.4 package which runs from the command line using Python scripts

Somatic Mutations
VCFtools (v1.14)
MutationalPatterns (v3.4.1)
Wald test of association of biological covariates and mutation counts (SNV, indels, fusions) was performed with glm function from base R (v4.1.3) specifying Poisson family distribution.
A cubic spline was built to adjust for read depth using the rcs function from the rms package (v6.3.0)
Pearson correlations: corr and cor.test functions from base R (v4.1.3)
Hypergeometric test: phyper function from base R (v4.1.3)
Principal components analysis (PCA): plotPCA from DESeq2 package and PCA gene loadings: prcomp function from base R (v4.1.3)
p-value correction: p.adjust function from base R (v4.1.3) correcting for false discovery rate (Benjamini and Hochberg, JR Stast. Soc., 1995)

Analysis results can be interactively viewed at https://oliverziff.shinyapps.io/als_genome_instability/

For manuscripts utilizing custom algorithms or software that are central to the research but not yet described in published literature, software must be made available to editors and reviewers. We strongly encourage code deposition in a community repository (e.g. GitHub). See the Nature Portfolio guidelines for submitting code & software for further information.

Data

Policy information about availability of data

All manuscripts must include a data availability statement. This statement should provide the following information, where applicable:
- Accession codes, unique identifiers, or web links for publicly available datasets
- A description of any restrictions on data availability
- For clinical datasets or third party data, please ensure that the statement adheres to our policy

Data availability statement:
iPSMN raw sequencing data used in this study are available in public repositories under accession numbers shown in Table 1.
Post-mortem raw sequencing data is accessible at GSE137810 and https://collaborators.nygenome.org/.
The accession numbers for the TDP-43 depletion raw sequencing data used are in Table S8.
Some raw data have restricted access (NeuroLINCS dbGaP Accession number: phs0001231.v2.p1; AnswerALS database). Granting access to these is beyond the control of the authors. Access can be obtained by applying to the relevant Data Access Committees. The authors declare that all other data supporting the findings of this study, including the source data for all figures, are publicly available without restrictions and also available from Supplement and the Github repositories.

Restricted data
NeuroLINCS RNA sequencing
Paper: Li et al. iScience 2021)
URL: http://neurolincs.org/
Raw data: https://www.ncbi.nlm.nih.gov/projects/gap/cgi-bin/study.cgi?study_id=phs001231.v2.p1#:~:text=The%20NeuroLINCS%20Center%20is%20part,drugs%20and%20other%20molecular%20factors.
Data access controlled (raw data): Study Accession number (dbGaP): phs0001231.v2.p1

AnswerALS DNA and RNA sequencing
Paper: Baxi et al., Nature Neuroscience, 2022
URL: https://www.answerals.org/
Data access:
Controlled (raw data):

Data can be downloaded with the custom AnswerALS download tool but requires a signed DUA to have full access.

Databases
Ensembl GRCh38.99 human reference genome can be accessed from https://www.ensembl.org/Homo_sapiens/Info/Index
The COSMIC database can be accessed with a free account without an application: https://cancer.sanger.ac.uk/cosmic
The REDIportal database of RNA editing sites can be accessed at http://srv00.recas.ba.infn.it/atlas/download.html

Human research participants

Policy information about studies involving human research participants and Sex and Gender in Research.

Reporting on sex and gender	n/a
Population characteristics	n/a
Recruitment	n/a
Ethics oversight	n/a

Note that full information on the approval of the study protocol must also be provided in the manuscript.

Field-specific reporting

Please select the one below that is the best fit for your research. If you are not sure, read the appropriate sections before making your selection.

☒ Life sciences ☐ Behavioural & social sciences ☐ Ecological, evolutionary & environmental sciences

For a reference copy of the document with all sections, see nature.com/documents/nr-reporting-summary-flat.pdf

Life sciences study design

All studies must disclose on these points even when the disclosure is negative.

Sample size	From the initial 16 datasets from 445 patients, we included 15 datasets that passed computational quality control comprising 429 iPSC derived motor neurons, of which 323 were from ALS patients and 106 from non-ALS controls. Postmortem ALS tissue was derived from the NYGC ALS cohort of 153 ALS patients and 80 controls, which were included to validate the findings of the iPSMN results. This represents the totality of publicly available samples and is a substantial advance on the power achieved in previous studies, which were typically limited to less than 10 patient samples especially in iPSC research (many iPSC studies have around n of 3). There is a large degree of variability and heterogeneity between samples from ALS patients and this is why we chose to maximize the sample size and power in this study in order to identify relatively modest effect sizes with smaller magnitudes than previously discovered.
Data exclusions	The Lee et al. dataset was excluded due to RNA library batch effects between ALS (Ribo-Zero) and control (polyA) samples as well as inadequate neuronal marker expression.<br>We excluded datasets that had not undergone a motor neuron differentiation protocol or failed iPSMN identity or RNA-seq quality control measures.<br>We excluded three control samples in AnswerALS that whole-genome sequencing revealed to have pathogenic ALS mutations. 4 Answer ALS iPSMNs from non-ALS motor neuron disease patients were excluded. NeuroLINCS consists of 3 distinct iPSC protocols (iMNs, diMNs and undifferentiated iPSCs; Table S1), of which only the iMN and diMN batches were included.<br>Postmortem samples from non-spinal cord sites were excluded as well as samples that failed quality control.
Replication	The signatures were produced from 429 samples from 10 different ALS mutations and sporadic ALS patients. To our knowledge there is no other iPSC derived motor neuron cohorts of ALS patients available other than the databases included in this study. We validated our approach by separating polyA and ribo-zero library preparations (Extended Data Fig. 8, Methods). We tested each genetic subgroup separately for the p53 pathway and TP53 transcription factor signature. We compared changes with postmortem spinal cord tissue from the extensive NYGC ALS cohort. Because of the costs involved with sequencing, individual samples were not sequenced multiple times on separate occasions. Individual datasets were anlaysed both together (integrated) and separate (independently) which successfully confirmed the results observed.
Randomization	No randomisation was performed - this was a descriptive study, not an experimental study.
Blinding	No blinding was undertaken - this was a descriptive study, not an experimental study.

Reporting for specific materials, systems and methods

We require information from authors about some types of materials, experimental systems and methods used in many studies. Here, indicate whether each material, system or method listed is relevant to your study. If you are not sure if a list item applies to your research, read the appropriate section before selecting a response.

Materials & experimental systems

n/a	Involved in the study
☒	☐ Antibodies
☒	☐ Eukaryotic cell lines
☒	☐ Palaeontology and archaeology
☒	☐ Animals and other organisms
☒	☐ Clinical data
☒	☐ Dual use research of concern

Methods

n/a	Involved in the study
☒	☐ ChIP-seq
☒	☐ Flow cytometry
☒	☐ MRI-based neuroimaging
